# Sources of Synaptic Input to Neurons in the Nucleus Accumbens Shell That Project to the Ventral Pallidum

**DOI:** 10.1002/cne.70081

**Published:** 2025-08-21

**Authors:** Shuanghong Li, Sa Li, Gilbert J. Kirouac

**Affiliations:** ^1^ Rady Faculty of Health Sciences University of Manitoba Winnipeg Manitoba Canada

**Keywords:** input, nucleus accumbens, rabies, RRID:AB_1549585, RRID:AB_2762833, thalamus, tracing, ventral pallidum, ventral subiculum

## Abstract

The shell of the nucleus accumbens (NAcSh) regulates motivation and reward via its dense projection to the ventral pallidum (VP). This ventral striatopallidal system has also been shown to regulate the activity of midbrain dopamine neurons and the release of dopamine in the NAcSh. The present study applied monosynaptic rabies tracing in the rat to quantify the brain‐wide sources of synaptic input to neurons in the medial NAcSh that project to the ventromedial VP. The ventral subiculum of the hippocampus (vSub) was the largest source of input cells to the NAcSh‐VP projection neurons. Anterograde tracing of vSub‐NAcSh projection neurons demonstrated that their fibers terminated densely in the NAcSh largely avoiding other regions of the striatum. Another relatively strong source of input cells included the anterior part of the paraventricular nucleus of the thalamus (aPVT). The CA1, lateral septal nucleus, VP, paratenial thalamic nucleus, bed nucleus of the stria terminalis, lateral preoptic area and dorsomedial nucleus of the hypothalamus were moderately strong sources of input neurons. The prefrontal cortex, amygdala, and the basolateral nucleus of the amygdala were found to be relatively weak sources of input. A lack of sex differences for all the sources of input identified indicates that there is no apparent sexual dimorphism in the afferents to the striatopallidal system. In summary, the vSub and the aPVT are the major sources of cortical and thalamic monosynaptic inputs to the NAcSh‐VP projection neurons where these inputs converge to regulate behavior and dopamine release in the NAcSh.

## Graphical abstract

Monosynaptic rabies tracing shows that the ventral subiculum and thalamic paraventricular nucleus are sources of input to a nucleus accumbens shell‐ ventral pallidum pathway involved in modulating dopamine neuron activity.

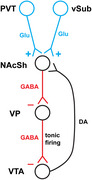

## 1 Introduction

The ventral striatopallidal pathway was conceptualized in the early 1980s as a neural system that converts limbic cortical signals into behavioral action (Floresco 2015; Groenewegen et al. 1993; Mogenson et al. 1980; Mogenson and Yang 1991; Nicola 2007; Pennartz et al. 1994; Root et al. 2015; Zahm 2000). The system is composed of the nucleus accumbens (NAc) and its projections to the ventral pallidum (VP) located immediately inferior and posterior to the NAc (Groenewegen et al. 1993; Heimer et al. 1991). In turn, the VP projects and influences motor circuits located in the hypothalamus and brainstem (Groenewegen et al. 1993; Nicola 2007; Pennartz et al. 1994; Swanson 2000). The NAc has been subdivided into an outer shell (NAcSh) and a central core (NAcC), with each of these subdivisions innervating specific regions of the VP (Groenewegen et al. 1993; Heimer et al. 1997; Heimer et al. 1991; Zahm and Heimer 1990). For instance, the medial NAcSh (mNAcSh) projects densely to the ventromedial region of the VP whereas the NAcC projects to a smaller dorsolateral region (Groenewegen et al. 1993; Heimer et al. 1991; Root et al. 2015).

Initial pharmacological evidence indicated that the NAcSh regulated locomotion and food intake via projection to the VP (Maldonado‐Irizarry and Kelley 1994; Mogenson et al. 1980; Mogenson and Yang 1991; Stratford and Kelley 1997; Stratford et al. 1998). Contemporary views based on a variety of experimental approaches support the view that the NAcSh suppresses or gates unconditioned behavioral responses by modulating the activity of VP and other descending targets (Floresco 2015; Nicola 2007; Pennartz et al. 1994; Root et al. 2015; Soares‐Cunha and Heinsbroek 2023). It is also understood the NAcSh's motivational function is closely associated with the release of dopamine (DA) within this region of the striatum (Nicola 2007; Pennartz et al. 1994; Root et al. 2015; Soares‐Cunha and Heinsbroek 2023). Other evidence demonstrates that activation of excitatory limbic cortical inputs to the NAcSh leads to disinhibition of DA neurons in the ventral tegmental area (VTA) that project back the striatum providing a means by which the NAcSh can modulate the amount of DA it receives (Floresco et al. 2003; Lodge and Grace 2011). It is apparent that this disinhibition occurs via activation of medium spiny neurons (MSN) that inhibit tonically active GABAergic neurons in the VP that project to the VTA (Floresco et al. 2003; Lodge and Grace 2011).

The hippocampal ventral subiculum (vSub), basolateral nucleus of the amygdala (BLA) and prefrontal cortex including the orbitofrontal, infralimbic, prelimbic, insular areas provide afferents that terminate in the mNAcSh (Groenewegen et al. 1987, 1997; Meredith and Wouterlood 1990; Wright et al. 1996; Wright and Groenewegen 1995, 1996). Thalamic sources of afferents originate predominantly from the paraventricular nucleus of the thalamus (PVT) where they terminate within the same region densely innervated by the vSub and DA neurons in the VTA (Berendse and Groenewegen 1990; Li and Kirouac 2008; Meredith and Wouterlood 1990; Parsons et al. 2007). In contrast, fibers from the BLA and prelimbic cortex overlap in smaller subregions of the NAcSh poorly innervated by the vSub, PVT and DA neurons (Groenewegen et al. 1996; Groenewegen et al. 1999).

**FIGURE 1:**
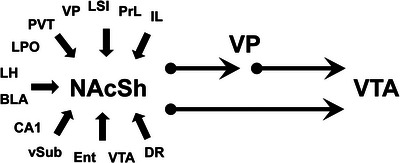
Schematic of the direct and indirect pathway from the NAcSh to the VTA with major known strong sources of afferents to the NAcSh as demonstrated using anterograde and retrograde tracer (Brog et al. 1993; Groenewegen et al. 1999). BLA, basolateral nucleus of the amygdala; CA1, field CA1 of the hippocampus; DR, dorsal raphe nucleus; Ent, entorhinal cortex; IL, infralimbic cortex; LH, lateral hypothalamus; LPO, lateral preoptic area; LSI, intermediate lateral septal nucleus; PrL, prelimbic cortex; PVT, paraventricular nucleus of the thalamus; VP, ventral pallidum; vSub, ventral subiculum; VTA, ventral tegmental area.

Retrograde tracing has also identified several other brain regions as sources of afferents to the mNAcSh including the VP, septal area, and entorhinal cortex (Brog et al. 1993; Groenewegen et al. 1999). Recent efforts to study the function of MSN involved using molecular approaches to probe MSN that express dopamine D1 or D2 receptors (Domingues et al. 2023; Soares‐Cunha et al. 2016; Thibeault et al. 2019). An underlying assumption of these approaches is that the D2‐expressing MSN (D2‐MSN) primarily project to the VP whereas D1‐expressing MSN (D1‐MSN) project to the VTA. Application of recombinant rabies virus (RV) tracing approaches to identify the source of monosynaptic inputs to D1‐MSN vs D2‐MSN in the NAc indicated that these neurons received similar combinations of input from diverse sources (Li et al. 2018). Interpretation of the latter investigation is limited by the fact that D2 receptor subtypes do not segregate uniquely on MSN that project to the VP (Kupchik et al. 2015). Indeed, it is likely that a proportion of D2‐MSN studied also projected to the midbrain while the D1‐MSN population that projected to the VP and could not be studied independently. Figure 1 summarizes the direct and indirect pathway from the NAcSh to the VTA in addition to the main sources of afferents to the NAcSh identified using traditional tracers. Traditional anterograde or retrograde tracers do not indicate if afferent fibers make synapses on specific projection neurons. The present investigation was done to identify sources of monosynaptic inputs to MSN that directly innervate the VP. An intersectional RV tracing approach was used to quantify the sources of these input to neurons in the mNAcSh that project to the medial VP (mVP) to identify the brain regions that are anatomically positioned to influence this critical system involved in regulating DA neurons and behavior.

## 2 Materials and Methods

### 2.1 Animals

A total number of 22 male and 21 female Sprague–Dawley rats (University of Manitoba vivarium) were injected with viral tracers of which a subset was analyzed. The rats weighed approximately 300 ± 10 g at the time of the injections and were housed on a 12:12 h light–dark cycle with food and water freely available. All experiments were carried out according to guidelines of the Canadian Council on Animal Care and approved by Research Ethics Review Board of the University of Manitoba.

### 2.2 Stereotaxic Injections

Rats were anesthetized with 1.5%–2.5% isoflurane, and the analgesic meloxicam (2 mg/kg, s.c.) was given at the time of surgery as well as 24 and 48 h after surgery. The animals were placed in a Stoelting stereotaxic frame, and a hand drill was used to expose the brain surface above the targeted regions on the right side of the brain. Microinjections of viral preparations and tracers were done using glass pipettes (approximately 30 µm tip diameter) connected to a pressure injection device (Picospritzer, Park Hannifin, Hollis, NH, USA) and administered over 10 min. The glass pipette was slowly withdrawn 10 min after the microinjection and the incision was sutured.

### 2.3 Intersectional Rabies Tracing

The intersectional RV tracing approach was used to quantify the inputs to mNAcSh neurons that project to the ventromedial VP. In a first surgical procedure, 500 nL of AAVrg‐Cre (AAVrg‐Syn1‐EBFP‐Cre; 7.6 × 10^12^ copies/mL; #51507‐AAVrg, Addgene, Cambridge, MA, USA) was microinjected in the ventromedial aspect of the VP (0.4 mm anterior, 1.5 mm lateral, 8.0 mm ventral; all coordinates are relative to bregma and bone surface; *n* = 10) combined with a 500 nL of Cre‐dependent helper AAV (AAV2/1 hSyn‐Flex‐TVA‐HA‐G, 1.0 × 10^11^ copies/mL; NTNU Viral Vector Core, Kavli Institute, Norway) microinjection in the medial aspect of the NAcSh (1.6 mm anterior, 1.0 mm lateral, 7.0 mm ventral) of the same animal. The helper AAV transduces the expression of the avian receptor TVA and rabies glycoprotein (G) in neurons that contain the Cre recombinase. The receptor TVA promotes entry of the pseudo‐typed RV with the envelop protein from avian sarcoma leucosis virus type A (EnvA), and the glycoprotein provides the G‐deleted rabies virus the critical component for transsynaptically infecting input cells (Wickersham et al. 2007). Two weeks later, in a second surgical procedure, a 500 nL of EnvA‐SADB19G‐deleted‐Rabies‐mCherry (RVdG‐mCherry, 1.0 × 10^10^ copies/mL; NTNU Viral Vector Core) was microinjected in the mNAcSh using the same coordinates as the helper AAV. The scalp was sutured and rats returned to their home cages for a week before perfusion with fixative. Control experiments were done by excluding either the AAVrg‐Cre injection in the VP (*n* = 3) or the helper AAV injections in the NAcSh (*n* = 3) prior to the RVdG‐mCherry injections.

### 2.4 Intersectional Anterograde Tracing

An intersectional anterograde tracing approach was used to examine fiber projections from vSub to the striatum. The approach involved transducing Cre recombinase in projection‐specific neurons using a retrograde AAV (AAVrg‐Cre) combined with a Cre‐dependent AAV that transduces GFP in neurons and fibers in the anterograde direction (Kirouac et al. 2022; Li et al. 2021). Subsequently, injection of the AAV‐GFP viral preparation in the vSub results in the transduction of vSub projecting neurons that contain Cre from the injection of AAVrg‐Cre in the mNAcSh. A microinjection of AAVrg‐Syn1‐EBFP‐Cre (500 nL) was made in the medial region of the NAcSh (*n* = 6) followed by a microinjection of an AAV9/Flex‐GFP (500 nL; 1.98 × 10^13^ GC/mL, Salk Institute Viral Vector Core, La Jolla, CA, USA) in the vSub (5.8 mm posterior, 2.8 mm medial, 8.8 mm ventral, 10° angle toward the lateral). The animals were allowed a 4‐week survival period before perfusion and the removal of the brain for sectioning.

### 2.5 Tissue Processing and Analysis

The rats were anesthetized with 10% chloral hydrate (430 mg/kg, i.p.) and perfused transcardially with 250 mL heparinized saline followed by 450–500 mL ice‐cold 4% paraformaldehyde in 0.1 M phosphate buffer (pH 7.4). The brains were removed and post‐fixed in the same fixative overnight and cryoprotected in phosphate buffered saline (PBS) containing 20% sucrose and 10% glycerin at 4°C for 48 h. Coronal sections of the brain were taken at 50 µm with a cryostat and stored in cryoprotectant until immunoreacted and/or mounted on glass slides. One sixth of the sections that contain the NAcSh for the rabies tracing experiments were preincubated in a blocking solution of PBS containing 5% donkey serum, 0.3% Triton X‐100, and 0.1% sodium azide for 1 h prior to a 48 h incubation in a primary antibody solution containing rabbit anti HA‐Tag (C29F4) monoclonal antibody (1:1000; Cell Signaling Technology Cat# 3724, RRID:AB_1549585) followed by a 2 h incubation in a solution containing a secondary Alexa‐Fluor Plus 488 donkey anti‐rabbit antibody (1:3000; Thermo Fisher Scientific Cat# A32790, RRID:AB_2762833). The HA‐Tag antibody was produced by immunizing animals with a synthetic peptide containing the influenza hemagglutinin epitope and the antibody has been shown using immunoprecipitation to detect recombinant proteins containing the HA epitope tag in transfected cells (Field et al. 1988). There was no labeling in controls that did not receive injections of the helper AAV or the AAVrg‐Cre. After a final set of rinses, sections were mounted and cover slipped using Fluromount‐G (Southern Biotech, Birmingham, AL, USA). Every sixth sections of the brain were mounted on glass slides for both the intersectional rabies and anterograde experiments and cover slipped for subsequent examination and analysis.

### 2.6 Image Capture and Data Analysis

Images showing the distribution of neurons and fibers were produced from compiled stacks of frames captured using Zeiss Axio Observer Z1 microscope equipped with Axiocam 503 mono camera. Image frames of starter cells were taken as stacks of 1 µm (Z axis) with a 20× objective lens set to cover a region of interest (X and Y axis) whereas images of input cells for figures were taken with a 10× lens with 2 µm stack. The exposure time was adjusted for individual color channel to optimize the captured images and kept consistent for the cases associated with a specific experiment. Images were processed using “Stitch” to fuse the X and Y tiles and then “Extended Depth of Focus” for the Z stacks (Zen Blue, Zeiss) to produce the tiled images. The contrast of the composite images was adjusted in Adobe Photoshop CS4 (Adobe Inc., San Jose, CA, USA) to produce the final images.

The number of starter cells in the NAcSh was quantified on sections captured at 300 µm intervals using Zeiss Axio Observer Z1 microscope with the methods described above. Starter cells in the NAcSh were identified as neurons with unambiguous co‐distribution of mCherry and HA within the same neuronal cell body. The soundness of the co‐distribution was further evaluated by switching between color channels to confirm that a clear overlap of the signal within the same soma. Neurons with co‐distribution of mCherry and HA were marked with a symbol for subsequent quantification and generation of the figure showing the distribution of starter cells in the NAcSh. The starter cells are the only neurons targeted by the RV and consist of NAcSh neurons that project to the VP. Input cells are the neurons that synapses on starter cells consisting of NAcSh‐VP projection neurons. The number of RVdG‐mCherry input cells for the different regions of the brain was quantified when there were 50 or more input cells over the series of sections collected for a particular region. Images of the regions were captured using a 4× objective on Olympus BX51 microscope equipped with a digital camera (SPOT Insight, Diagnostic Instruments Inc, Sterling Heights, MI, USA). Input cells were marked with a symbol on images using Adobe Photoshop followed by quantification using ImageJ (Fiji).

A figure showing the stereotaxic distribution of the input cells to NAcSh‐VP projecting neurons was generated by importing the images with the marked symbols into Adobe Illustrator CS4 (Adobe Inc.) and overlaying them with image files of the appropriate level of the NAcSh in a digital atlas of the rat brain (Paxinos and Watson 2009). The boundaries of brain nuclei and fiber bundles from the atlas images were adjusted to correspond to the microscopic image. The merged images were exported to Adobe Photoshop and a symbol to indicate the location of a labeled neuron was manually placed on the image. The final image files of individual stereotaxic levels were used to generate the figures displaying labeled neurons on selected stereotaxic levels of the brain. Images of the vSub and the NAc were taken to show the location of neurons and fibers expressing GFP in the NAcSh, respectively.

### 2.7 Statistical Analyses

The data for the RV tracing experiments were analyzed using a two‐tail *t*‐test to examine sex differences in the number of starter cells. The number of input cells and proportion of input cells per brain area were analyzed using a two‐way ANOVA followed by Tukey's multiple comparison tests using GraphPad Prism version 9.5.1 for Windows (GraphPad Software, San Diego, California USA). An adjusted value of *p* < 0.05 was considered significant and the data are presented as mean ± SEM.

## 3 Results

### 3.1 Intersectional Rabies Tracing

**FIGURE 2:**
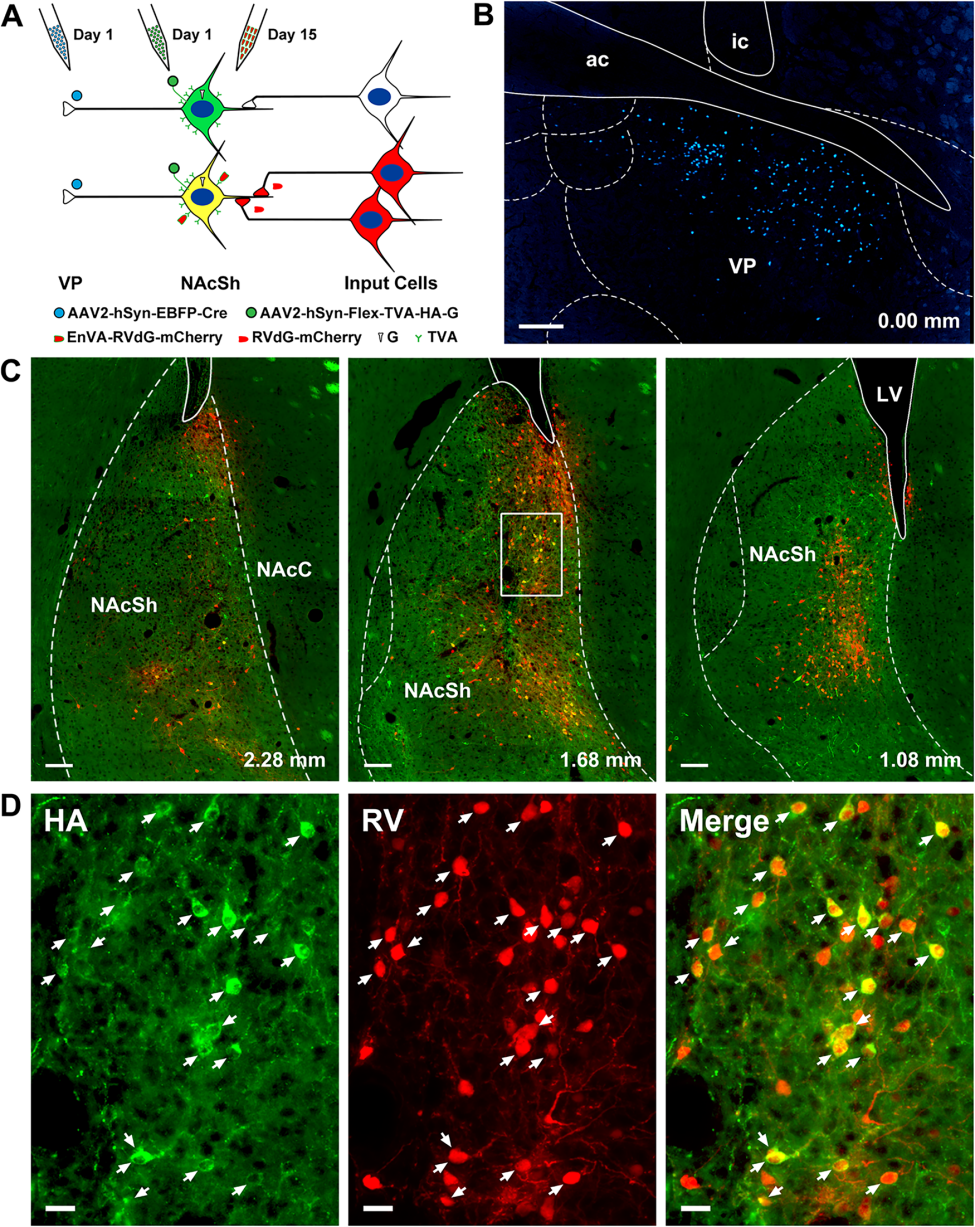
Schematic of the intersectional rabies tracing approach and resulting examples of starter cells in the NAcSh. (a) Schematic of the viral injections associated with the intersectional rabies tracing approach. (b) Representative example of an AAVrg‐EBFP‐Cre injection in the VP in one case. (c) Examples of the resulting starter cells in the NAcSh with targeting the helper AAV and RV to NAcSh‐VP projection neurons shown at lower magnification. (d) Higher magnification of the inset shown in row above with white arrows indicating starter cells. Starter cells are defined by the presence of green immunofluorescence for hemagglutinin (HA) transduced by the helper AAV and the red mCherry transduced by the RV. ac, anterior portion of the anterior commissure; ic, internal capsule; LV, lateral ventricle; NAcSh, nucleus accumbens shell; NAcC, nucleus accumbens core; VP, ventral pallidum. Numbers at the bottom indicate distance from bregma. Scale bars: 200 µm for B; 100 µm for C and 20 µm for D.

Figure 2 shows a schematic of the intersectional RV tracing approach and starter cells in the mNAcSh in one case with injection of the AAVrg‐Cre in the mVP. Starter cells were defined by the presence of mCherry transduced by RVdG and HA by the helper AAV (Figure 2c and d). The cases analyzed were selected based on the presence of starter cells located primarily in the mNAcSh (Figure 2c) and the presence of a micropipette tract and EBFP positive neurons in the mVP (Figure 2b). Most of the starter cells were in the dorsomedial region of the NAcSh with some observed in the adjacent NAcC. The number of starter cells in the NAcC comprised only a small fraction of the total number of starter cells identified. The number of starter cells for cases involving female (699.3 ± 189.3 cells, *n* = 6) and male (672.5 ± 196.4 cells, *n* = 4) was not statistically different (*t*_(8)_ = 0.098, *p* = 0.92).

**FIGURE 3:**
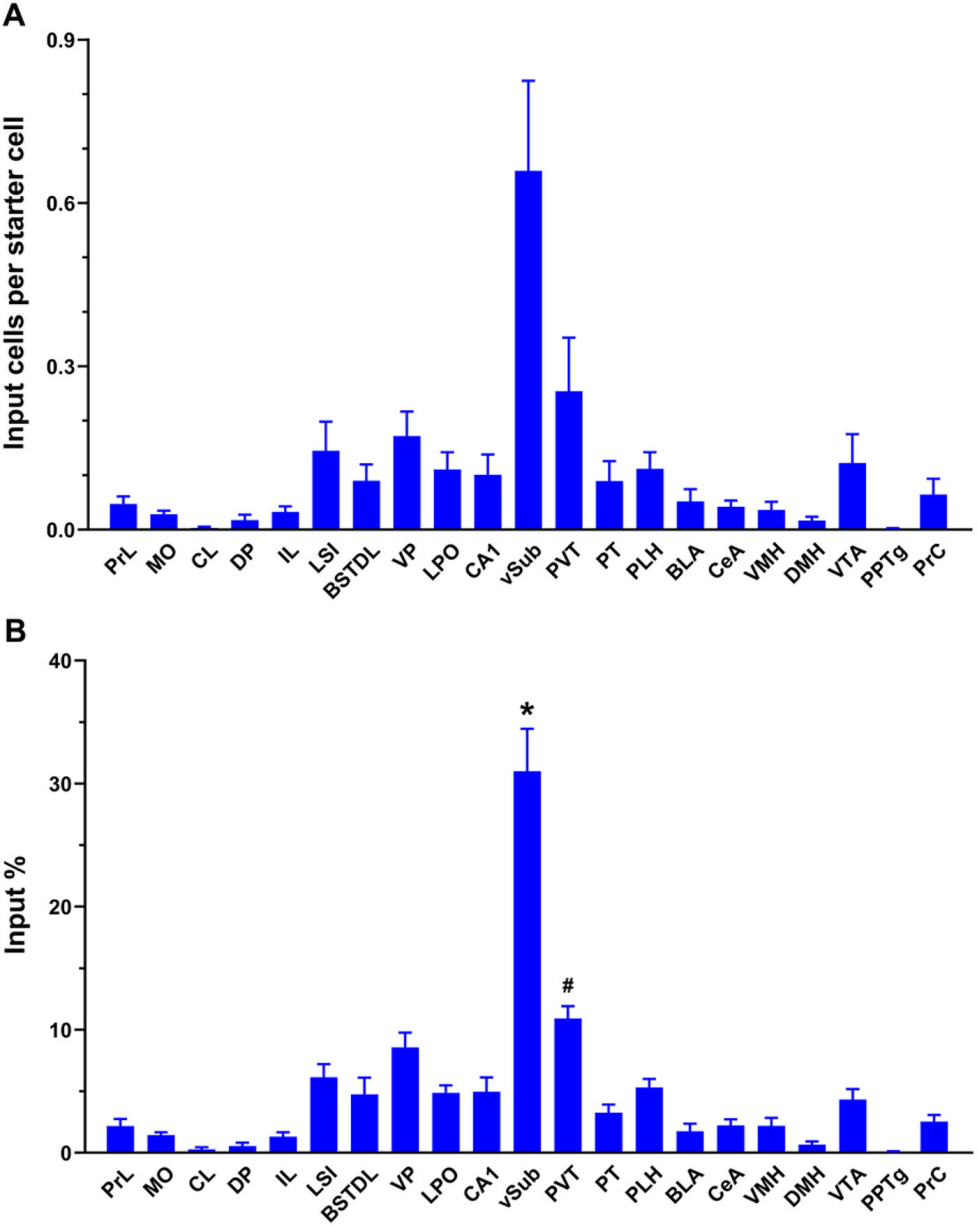
Rabies input cells to the NAcSh‐VP projection neurons. (a) Number of rabies input cells per starter cell by input region (*n* = 10). (b) Percentage of input cells by input region. BLA, basolateral nucleus of the amygdala; BSTDL, dorsolateral bed nucleus of the stria terminalis; CA1, field CA1 of the hippocampus; CeA, central nucleus of the amygdala; CL, claustrum; DP, dorsal peduncular cortex; DMH, dorsal nucleus of the hypothalamus; IL, infralimbic cortex; LPO, lateral preoptic area; LSI, intermediate lateral septal nucleus; MO, medial orbital nucleus; PLH, peduncular part of the lateral hypothalamus; PPTg, pedunculopontine tegmental nucleus; PrC, precommissural nucleus; PrL, prelimbic cortex; PT, paratenial nucleus of the thalamus; PVT, paraventricular nucleus of the thalamus; VMH, ventromedial nucleus of the hypothalamus; VP, ventral pallidum; vSub, ventral subiculum; VTA, ventral tegmental area. *^#^ *p* < 0.05, * vSub compared to all the other structures; ^#^ PVT compared to all structures but LSI, VP, LPO, CA1, PLH and VTA.

Input cells were located on the same side as the viral injections associated with the starter cells. The number of input cells per starter cell was found to vary by the areas of the brain quantified (Figure 3a). The two‐way ANOVA indicated a main effect for input cells by area (*F*_(20,168)_ = 7.519, *p* < 0.0001) with no interaction between area and sex (*F*_(20,168)_ = 2.493, *p* = 0.996). We were interested in determining the proportion of input cells originating from the various regions of the brain sending afferents to NAcSh‐VP neurons. Accordingly, input cell data were converted to percentage of input per starter cells (Figure 3b). The two‐way ANOVA indicated a main effect for percentage of input cells by area (*F*_(20,168)_ = 37.90, *p* < 0.0001) with no interaction between area and sex (*F*_(20,168)_ = 0.6073, *p* = 0.9037). There were a few notable differences as indicated by the post hoc analysis. The vSub was undeniably the largest source of input cells where this area was significantly different from several other areas quantified including the PVT, VP, and LSI (Figure 3a and b). The vSub comprised more than 30% of input cells to the NAcSh‐VP projection neurons whereas the PVT and VP each contributed approximately 10% of the input cells. The anterior aspect of the PVT (aPVT) was an especially strong source of input cells, and this thalamic nucleus was a significantly stronger source than several other brain areas including the thalamic paratenial nucleus (PT).

**FIGURE 4:**
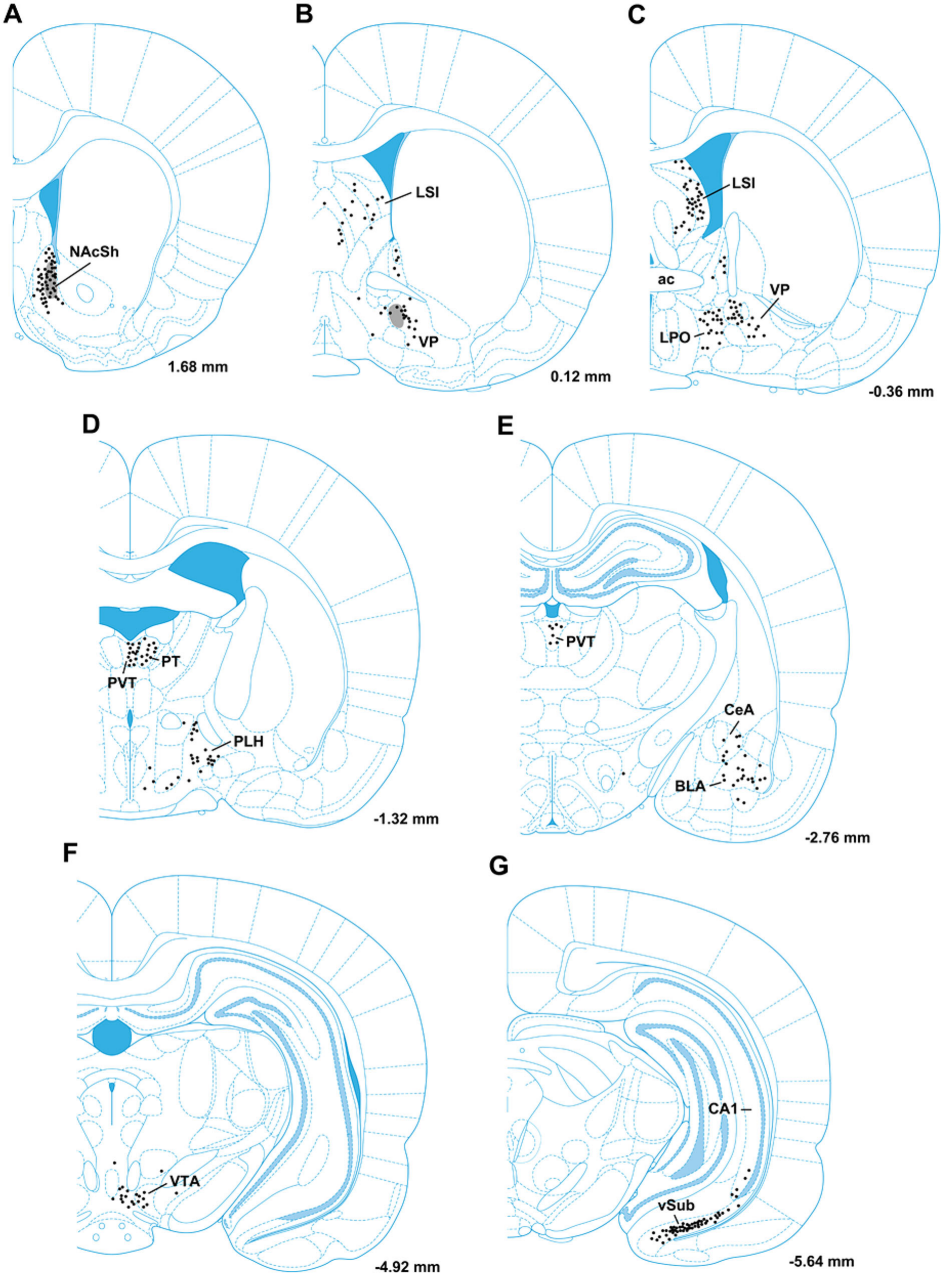
Location of rabies input cells in the forebrain to NAcSh‐VP projection neurons. The general location of the starter cells in the NAcSh is illustrated as gray shading with dots (a) and the location of the AAVrg‐Cre in the VP at this stereotaxic level is illustrated as gray sphere (b). Each dot represents a single input cell and is indicated on illustrations modified from a digital stereotaxic atlas of the rat brain (Paxinos and Watson 2009). ac, anterior portion of the anterior commissure; BLA, basolateral nucleus of the amygdala; CA1, field CA1 of the hippocampus; CeA, central nucleus of the amygdala; NAcSh, nucleus accumbens shell; LPO, lateral preoptic area; LSI, intermediate lateral septal nucleus; PLH, peduncular part of the lateral hypothalamus; PT, paratenial nucleus of the thalamus; PVT, paraventricular nucleus of the thalamus; VP, ventral pallidum; vSub, ventral subiculum; VTA, ventral tegmental area. Numbers at the bottom indicate distance from bregma.

**FIGURE 5:**
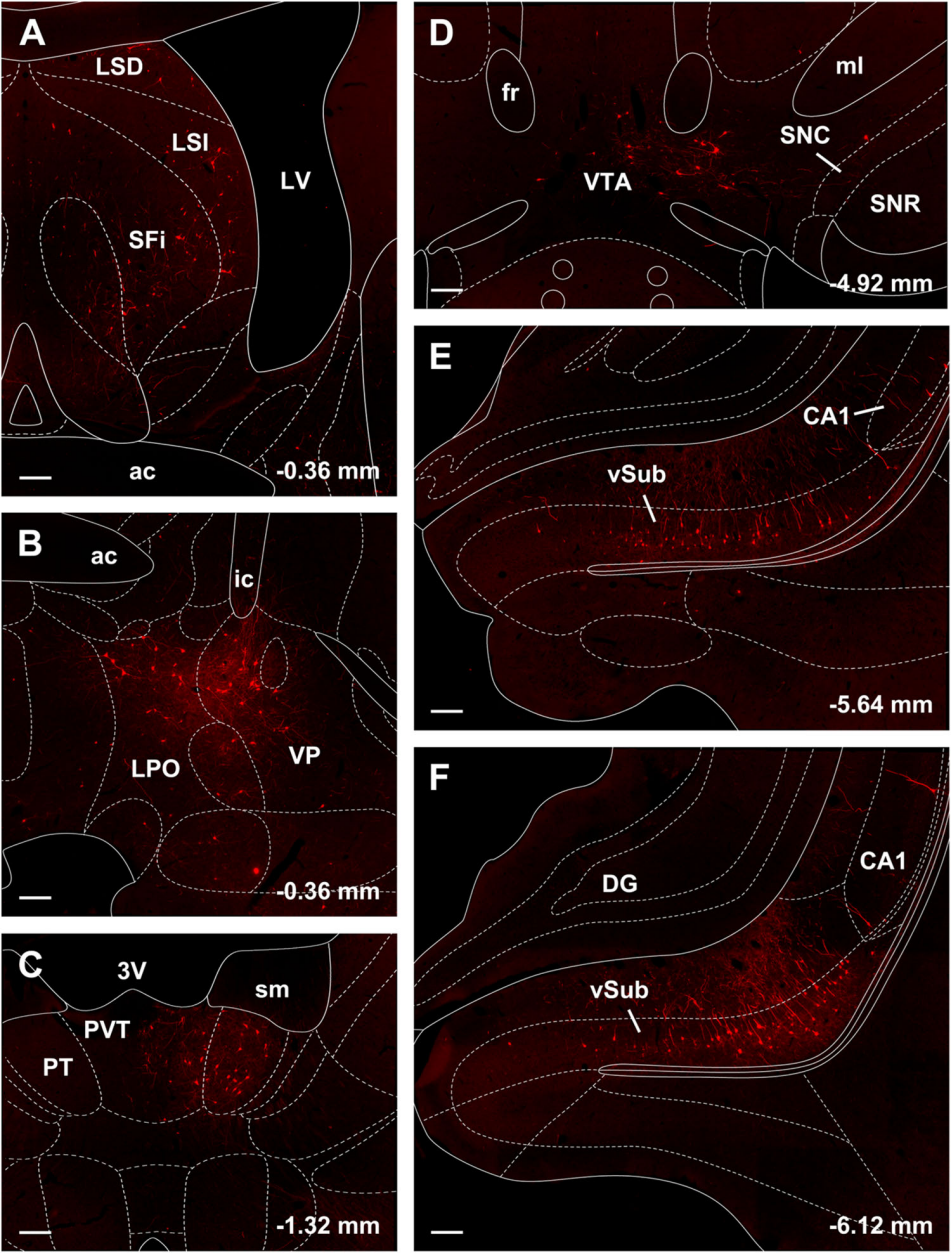
Images of regions of the brain containing many rabies input cells to NAcSh‐VP projection neurons. The images are from the same case shown in Figures 2 and 4. 3 V, third ventricle; ac, anterior commissure; CA1, field CA1 of the hippocampus; DG, dentate gyrus; fr, fasciculus retroflexus; ic, internal capsule; LPO, lateral preoptic area; LSD, dorsal lateral septal nucleus; LSI, intermediate lateral septal nucleus; ml, medial lemniscus; PT, paratenial nucleus of the thalamus; PVT, paraventricular nucleus of the thalamus; SNC, substantia nigra compact part; SNR, substantia nigra reticular part; SFI, septofimbrial nucleus; sm, stria medullaris of the thalamus; VP, ventral pallidum; vSub, ventral subiculum; VTA, ventral tegmental area. Numbers indicate distance from bregma. Scale bars: 200 µm.

Figures 4 and 5 show the distribution of input cells in areas of the brain where most of the labeled neurons were found in the case associated with starter cells shown in Figure 2. Input cells were scattered in the septal nuclei with the majority of these being found in the LSI (Figures 4c and 5a). Cells were also observed in the VP, bed nucleus of the stria terminalis, and lateral preoptic area (Figures 4b and c and 5b). The aPVT and the PT of the dorsal midline thalamus consistently contain input cells (Figures 4d and e and 5c). Labeled neurons were located only in the aPVT with the posterior thalamus devoid of input cells. A scattering of input cells was observed in the amygdala concentrated primarily in the central nucleus and BLA (Figure 4e). A few input cells were consistently observed in the VTA of each section examined (Figures 4f and 5d). The greatest number of input cells was found in the vSub adjacent to the CA1 field (Figures 4g and 5e and f).

### 3.2 Intersectional Anterograde Tracing

**FIGURE 6:**
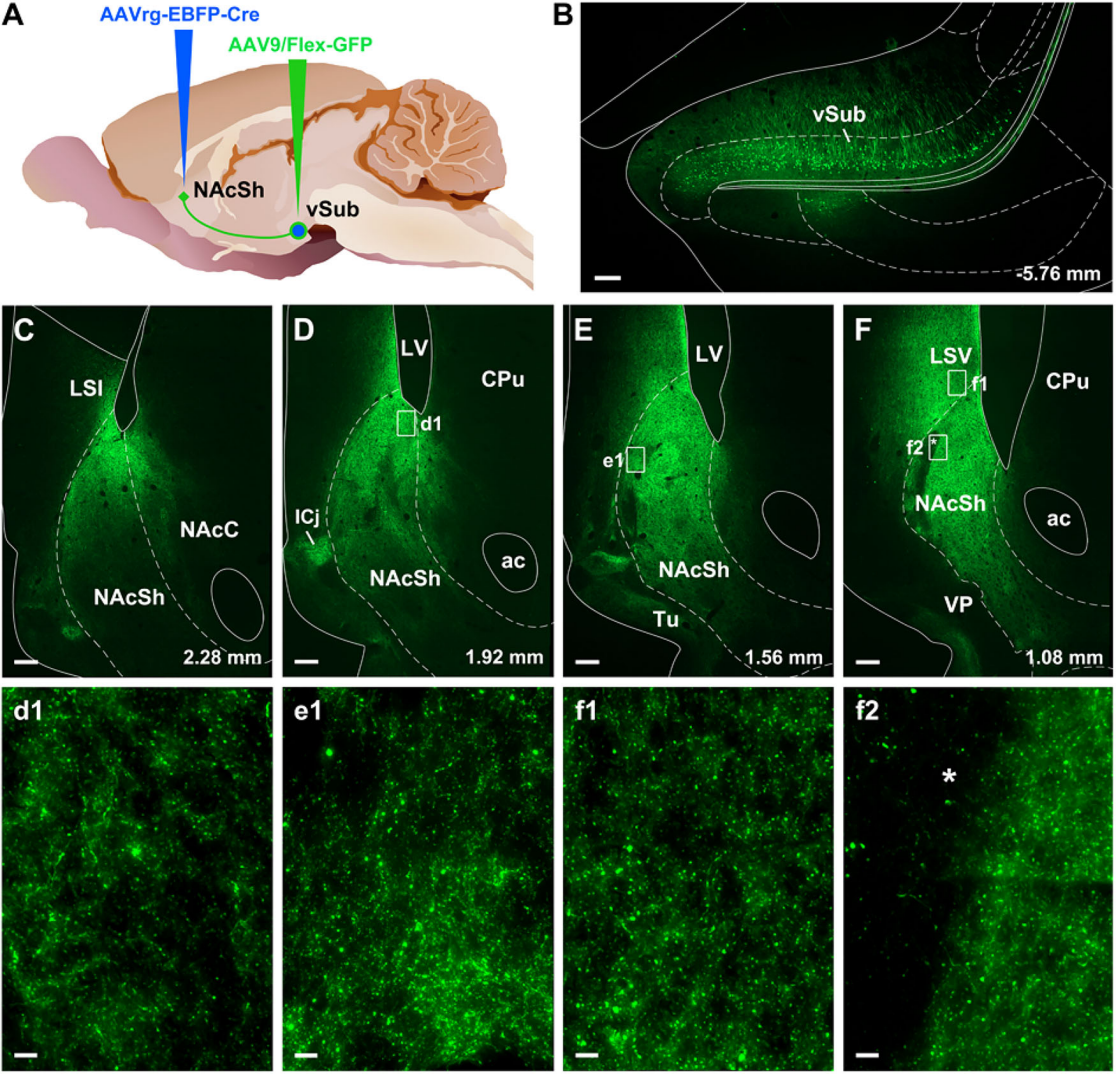
Intersectional anterograde tracing of vSub neurons projecting to the NAcSh. (A) Schematic of the anterograde tracing approach. (B) GFP labeled neurons in the vSub projecting to the NAcSh. (C‐F) Dense GFP labeled fibers were observed in the dorsomedial NAcSh and the intermediate and ventral lateral septal nucleus (LSI/LSV). (d1‐f2) Higher magnification of fibers in areas outlined by insets shown in panels D–F. Dense GFP labeled fibers were observed in medial NAcSh and immediately adjacent NAcC and islands of Calleja. Note the * marks subregions of the NAcSh known to receive dense DA innervation. ac, anterior portion of the anterior commissure; CPu, caudate putamen; iCj, islands of Calleja; LSI, intermediate lateral septal nucleus; LSV, ventral lateral septal nucleus; LV, lateral ventricle; NAcC, core of the nucleus accumbens; NAcSh, shell of the nucleus accumbens; Tu, olfactory tubercle; VP, ventral pallidum; vSub, ventral subiculum. Numbers indicate distance from bregma. Scale bars: 200 µm; 10 µm for the inset.

Neurons transduced by the AAV‐GFP injections in the vSub were localized throughout the vSub (Figure 6b) like what has be reported after injections of retrograded tracers in the NAcSh (Brog et al. 1993). A dense plexus of fibers was observed in the dorsomedial NAcSh and the ventral part of the lateral septal nucleus (LSV) immediately above the septal pole of the NAcSh (Figure 6f). The location of the vSub fibers corresponded to the region of the NAcSh where most starter cells were in the RV tracing experiments. Fibers were also observed immediately adjacent NAcC and islands of Calleja. More weakly labeled fibers could also be seen in the olfactory tubercle while largely avoiding the VP interdigitated between the olfactory tubercle and the NAcSh.

## 4 Discussion

Monosynaptic rabies tracing was used to examine the sources of synaptic input to NAcSh‐VP projecting neurons. These experiments demonstrate that these neurons are anatomically positioned to integrate signals from other cells located in several regions of the brain. The vSub is undoubtedly the major source of input neurons comprising over 30% of all the input cells identified in the present investigation. Another relatively strong source of input cells is the aPVT where these cells were found predominantly in the anterior aspect of this midline thalamic nucleus. Surprisingly, areas of the prefrontal cortex and the BLA comprised a relatively small proportion of all input cells. Anterograde tracing of vSub‐NAcSh projecting neurons establishes that vSub neurons primarily project to the NAcSh while avoiding other areas of the ventral striatum. Evidence from the anterograde and rabies tracing experiments points to the importance of the vSub as the major source of afferent input to NAcSh‐VP projection neurons.

Similar findings were reported when the monosynaptic rabies approach was targeted to D1 and D2‐MSN in the NAcSh (Li et al. 2018). The vSub was reported to be the largest source of input cells to both D1 and D2‐MSN, many of which likely projected to the VP based on what is known about the projections of these neurons (Kupchik et al. 2015; Zhou et al. 2003). It should be appreciated that NAcSh‐VP projecting neurons comprise MSN that express either D1 or D2 receptors or a combination of these receptors (Kupchik et al. 2015). There were some apparent differences between the results of our experiments and the previous study targeting the D2‐MSN. For instance, the relative proportion of all input cells originating in the LSI, VP, hypothalamus, BST, and CeA was noticeably greater in our experiments than what was reported for D2‐MSN. The fact that the vSub has been identified as the strongest source of synaptic input to NAcSh‐VP neurons combined with similar observation targeting the D2‐MSN should be taken as compelling evidence for the importance of neurons in this ventral hippocampal region in the excitation of this critical descending projection system. A similar argument can be made for the PVT even though the number of input cells was somewhat less than those located in the vSub. The fact that the vSub makes up a larger area of neural tissue than does the aPVT and some of the other areas quantified in the present study should be seen as a factor contributing to the high number of input cells observed in the vSub. Consequently, our results combined with the study targeting D2‐MSN in the NAcSh provide consensus for the vSub and PVT being the brain regions with the most direct influence on NAcSh‐VP projecting neurons.

The RV transfers across synaptic connections (Beier 2019; Rogers and Beier 2021; Wickersham et al. 2007) but it is not known if the virus transfers with similar efficiency across GABAergic or glutamate synapses (Beier 2019; Rogers and Beier 2021). It is also clear that the virus transfers poorly across monoaminergic synapses as evidenced by the comparative weak labeling of DA input cells when rabies is targeted to MSN in the striatum (Li et al. 2018; Wall et al. 2013). Relatively few input cells were observed in the VTA in the present study or when the RV is targeted to D1‐ or D2‐MSN in the NAcSh despite a dense innervation from DA neurons in the VTA (Ikemoto 2007). It is also important to appreciate that the transfer efficiency of the RV may be in part dependent on afferent fiber activity, or the enhanced synaptic strength associated with neuroplasticity (Beier et al. 2017; Rogers and Beier 2021). Indeed, the input organization observed in present investigation would likely be different if the rats had been exposed to conditions associated with changes in synaptic strength. The intersectional approach used in the present article resulted in starter cells predominantly located in the NAcSh combined with smaller numbers seen in the adjacent NAcC and septal nuclei. An intersectional anterograde tracing approach was also used to examine the specificity of the vSub projection to the mNAcSh. This approach resulted in labeling of cells primarily located in the vSub in a pattern consistent with the location of input cells in the rabies tracing experiments. Moreover, the fibers from the vSub labeled with this approach overlapped the region of the mNAcSh where the rabies starter cells were located.

It is generally recognized that MSN in the NAcSh densely innervate the VP where they release GABA to inhibit tonically active VP neurons that control behavior via other descending pathways or thalamocortical feedback circuits (Root et al. 2015; Smith et al. 2009). There is a wealth of experimental evidence supporting a role for NAcSh afferents to the VP in the modulation of behavior (Root et al. 2015). Much of this evidence comes from injections of pharmacological agents that are postulated to mimic the effects of changes in activity of NAcSh afferents in the VP. The evidence points to the view that many of the functions ascribed to the NAcSh are mediated via its projections to the VP. This includes behaviors related to reward, aversion, food consumption in addition to modulation of motor reflexes and processing of cognitive information (Root et al. 2015; Smith et al. 2009). The identification of sources of inputs to NAcSh‐VP projection neurons is an initial first step in understanding which afferents may be involved in regulating some of these behavioral responses. Abundant input cells in the vSub point to the potential importance of this limbic cortical region regulating a host of responses mediated by NAcSh‐VP pathway. The vSub is in a focal position to direct the output of the hippocampus and has been linked to processing of spatial information, memory, and motivation (Aggleton and Christiansen 2015; Matsumoto et al. 2019; O'Mara et al. 2009). Pyramidal cells in the vSub exert an excitatory effect on MSN in the NAcSh (Goto and O'Donnell 2001; O'Donnell and Grace 1995), an effect that would have a potentially broad influence on behavior via GABAergic inhibition of the VP. For example, optogenetic stimulation of vSub afferents to the NAcSh increases locomotion whereas inhibition reduces effort expenditure to bar press for food (Lindenbach et al. 2022). The PVT, which was also identified as a strong source of input cells to the NAcSh‐VP neurons, has similarly been linked to the regulation of motivation via its projection to the NAcSh (Iglesias and Flagel 2021; McGinty and Otis 2020; Millan et al. 2017; Penzo and Gao 2021). The activity of PVT neurons and their afferents to the NAc is enhanced by motivationally salient stimuli and states (Beas et al. 2024; Choi et al. 2019; Ma et al. 2021; Zhu et al. 2018). Potentiation of the PVT input to the D2‐MSN that presumably innervate the VP mediates conditioned place avoidance to morphine withdrawal (Zhu et al. 2016).

While there is general understanding that the prefrontal cortex is critically involved in cognition and behavior (Euston et al. 2012; Kesner and Churchwell 2011), this input accounted for a minor proportion of all synaptic input to the NAcSh‐VP neurons. This included the infralimbic and prelimbic areas considered strong sources of afferents to the NAcSh based on anterograde tracing approaches (Vertes 2004; Wright and Groenewegen 1995). The BLA, another cortical‐like region known to regulate behavior via projections to the NAc (Keefer et al. 2021), was similarly found to be a relatively minor source of inputs to NAcSh‐VP neurons. A small but consistent number of input cells were observed in the PT immediately lateral to the aPVT. The function of this thalamic nucleus is poorly understood even if it provides a relatively dense projection to the NAc (Vertes and Hoover 2008). We found that the intermediate lateral septal nucleus (LSI) and the ventral hippocampal CA1 accounted for a moderate proportion of input cells. It is notable that the vSub is interconnected with both the LSI and CA1 (Aggleton and Christiansen 2015; Besnard and Leroy 2022). The LSI is involved in the regulation of social, maternal and other motivated behavior (Besnard and Leroy 2022; Menon et al. 2022; Puska et al. 2024) but if and how a complex circuit between the vSub, CA1, LSI and NAcSh‐VP is involved in behavioral regulation remains to be examined. It was also notable that vSub‐NAcSh projecting neurons also provided a dense innervation of the LSV, a portion of the lateral septal nucleus linked to the regulation of stress, feeding and emotions (Wang et al. 2022; Xu et al. 2019).

It is difficult to speculate about the potential functional role of other lesser sources of input to NAcSh‐projection neurons considering how understudied these afferents to the NAcSh are compared to the cortical and thalamic inputs. The VP and lateral preoptic area were found in the present study to be consistent sources of input cells to NAcSh‐VP neurons. Both regions are recipients of descending afferents from the NAcSh (Heimer et al. 1991; Zahm et al. 1999a) and recent evidence demonstrates that both the VP and preoptic area modulate locomotion and other motivational responses (Reichard et al. 2019; Subramanian et al. 2018). Whether these effects are mediated via ascending projection to the NAcSh or other projections remains to be determined. The dorsolateral region of the bed nucleus of the stria terminalis and the lateral region of the central nucleus of the amygdala also contained a small number of input cells. This latter observation is consistent with previous descriptions of projections from these regions of the extended amygdala to the NAcSh (Zahm 1998).

A main purpose of the rabies tracing experiments was to identify the major sources of afferent synaptic input that can influence NAcSh‐VP projection neurons to better understand how the NAcSh regulates the activity of DA neurons in the VTA. Both the vSub and aPVT were identified as significantly greater sources of input cells to these projection neurons. There is good evidence that the activity of VTA DA neurons is controlled by top‐down mechanisms (Grace and Gomes 2019; Lodge and Grace 2011). Tonically active GABA neurons in the VP densely innervate the VTA where they act to keep DA neurons silent or firing at a slow tonic rate (Floresco et al. 2003; Gomes and Grace 2021; Lodge and Grace 2011; Sonnenschein et al. 2020). Indeed, inactivation of the VP produces a lasting increase in DA levels in the NAcSh as measured with microdialysis (Floresco et al. 2003). There is also evidence that a projection from the ventral hippocampus to the NAcSh regulates the activity of DA neuron in the VTA and the release of DA in the NAcSh. More specifically, activation of the ventral hippocampus at the interface of vSub and CA1 using infusions of NMDA increases the number of tonically active DA neurons in the VTA (Floresco et al. 2001; Floresco et al. 2003; Lodge and Grace 2006; Perez and Lodge 2018). Other evidence demonstrates that the effect of ventral hippocampal activation on DA neuron tonic activity is mediated by a projection from NAcSh to the VP since the effect is prevented by inactivation of the VP with bicuculline (Floresco et al. 2003). Infusions of NMDA or electrical stimulation of the ventral hippocampus also increases DA levels in the NAcSh via activation of its glutamate afferent fibers to the NAcSh (Blaha et al. 1997; Floresco et al. 2003; Forster and Blaha 2003). The observation that the vSub is the major source of input cells to NAcSh‐VP projecting neurons suggests that this is the population of ventral hippocampal neurons likely regulating the activity of DA neurons. Afferents from the PVT to the NAcSh have similar effects on DA neuron activity and release. For instance, activation of the PVT with NMDA infusions increases the number of tonically active DA neuron in the VTA via its glutamate afferents to the NAcSh (Perez and Lodge 2018) whereas electrical stimulation of the PVT increased DA levels in the NAcSh (Parsons et al. 2007). Other evidence demonstrates that projections from the vSub and PVT to the NAcSh function cooperatively to modulate the activity of VTA DA neurons (Perez and Lodge 2018). Indeed, the later paper provides electrophysiological evidence of convergence of vSub and PVT input to the same neuron in the NAcSh. Whether other prominent sources of input cells also contribute to the regulation of DA neurons via modulation of a NAcSh‐VP system remains to be determined.

### 4.1 Unresolved Questions

The NAcSh‐VP pathway is critically involved in regulating behavior and the activity of DA neurons in the VTA that project back to the ventral striatum. It is apparent from the rabies tracing results that these NAcSh‐VP neurons integrate inputs from several other brain regions in addition to limbic cortical areas and the thalamus. As discussed above, both the vSub and aPVT have been shown to regulate both behavior and DA neurotransmission via their glutamate afferents to the NAcSh. The contribution of other major sources on synaptic input is not known. It is also an open question if the influences of the vSub and aPVT on behavior and DA are functionally linked.

## Author Contributions

GJK, SHL, and SL designed the experiments and wrote the first draft of the manuscript. SHL and SL carried out the research and analysis and were involved in preparing all aspects of the manuscript.

## Conflicts of Interest

No conflict to declare.

## Peer Review

The peer review history for this article is available at https://publons.com/publon/10.1002/cne.70081

## Data Availability

The data that support the findings of this study are available on request from the corresponding author. The data are not publicly available due to privacy or ethical restrictions.
